# Supplementary material for: CATASAN Is a New Anti-Biofilm Agent Produced by the Marine Antarctic Bacterium Psychrobacter sp. TAE2020
Source: Mar Drugs. 2022 Nov 27;20(12):747. doi: 10.3390/md20120747 (PMC9785100; doi:10.3390/md20120747)
Supplement: Supplementary file 1 [file marinedrugs-20-00747-s001.zip › marinedrugs-2026376-supplementary.pdf]

Supplementary Material

CATASAN: A new anti-biofilm agent produced by the marine Antarctic bacterium *Psychrobacter* sp. TAE2020

Caterina D' Angelo,[1] Angela Casillo,[1] Chiara Melchiorre[2], Maria Michela Corsaro[1], Andrea Carpentieri[1], Maria Luisa Tutino,[1] Ermenegilda Parrilli[1]*

*** Correspondence:** erparril@unina.it (E. Parrilli); Tel: +39-081674003; fax:+39-081674113

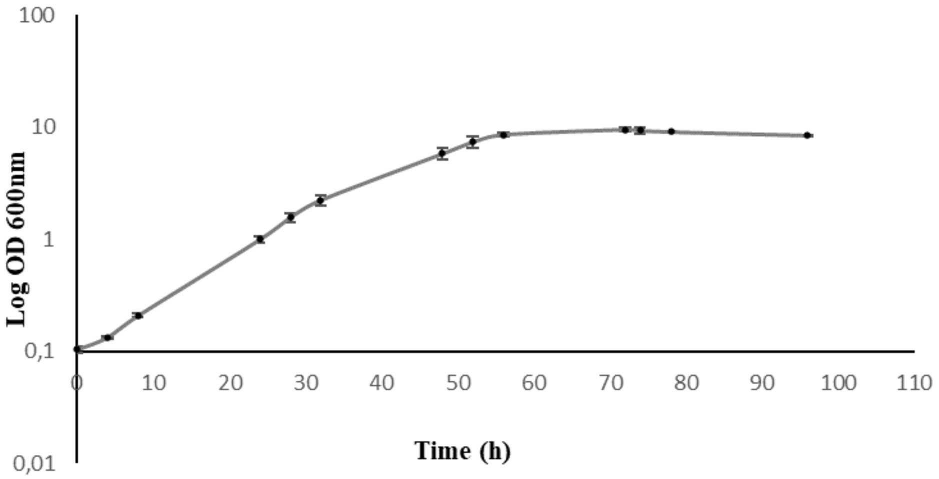

Figure S1. *Psycrobacter sp.* TAE2020 cell growth curve at 15°C in L-Glutamate culture medium.

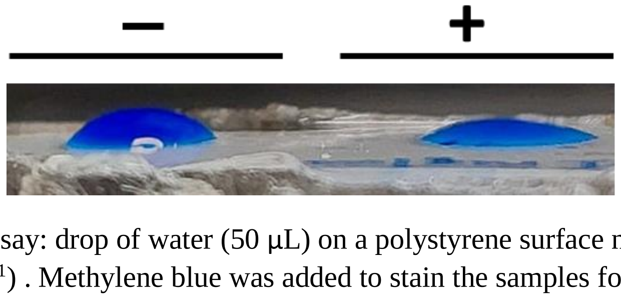

Figure S2. Drop collapse assay: drop of water (50 µL) on a polystyrene surface non-coated (-) and coated (+) with CATASAN (1 mg mL^{-1}) . Methylene blue was added to stain the samples for photographic purposes and had no influence on the shape of the droplets.
